# Temporal changes in geographical disparities in alcohol-attributed disease mortality before and after implementation of the alcohol tax policy in Taiwan

**DOI:** 10.1186/1471-2458-12-889

**Published:** 2012-10-22

**Authors:** Chih-Ming Lin, Tzai-Hung Wen

**Affiliations:** 1 Department of Healthcare Information and Management, Ming Chuan University, Taoyuan, Taiwan; 2 Department of Geography, National Taiwan University, Taipei, Taiwan

**Keywords:** Alcohol-attributed disease, Mortality, Alcohol taxation, Spatial analysis

## Abstract

**Background:**

Taxation of alcohol-containing products may effectively reduce alcohol consumption. However, whether alcohol taxation may lead to a decrease in alcohol-attributed disease mortality (ADM) remains unclear. The objective of this study was to assess the effect of alcohol tax policy in 2002 in Taiwan on temporal changes in geographical disparities in ADM before and after implementation of the policy.

**Methods:**

Local spatial statistical methods were used to explore the geographic variations in ADM rates and identify statistically significant clusters among townships.

**Results:**

Our results indicate that the areas with the highest rates of ADM (127-235 deaths per 100,000 people) were located in mountainous regions, and the areas with the lowest rates of ADM (less than 26 deaths per 100,000 people) were clustered in the most populated areas. The areas where the rates of ADM significantly declined after alcohol taxation was initiated were clustered in the central, southwest and northeast parts of the country.

**Conclusions:**

This study provides evidence of a township-level relationship between the reduction of ADM and alcohol taxation in Taiwan.

## Background

In most parts of the world, the health burden (morbidity, mortality, and disability) related to alcohol consumption is substantial. The World Health Organization (WHO) estimated that the use of alcohol leads to approximately 2.3 million premature deaths per year worldwide (3.7% of total global mortality) and is responsible for 4.4% of the global burden of disease [1]. In US, approximately 85,000 deaths per year are associated with drinking, including unintentional or intentional injuries and a range of diseases [2,3]. The alcohol-associated disease burden is closely related to the average volume of alcohol consumption, and the disease burden is heaviest among the poor and those who are marginalized by society [4].

Some studies also have demonstrated that alcohol prices or taxes are associated with morbidity or mortality [5-12]. The relationship between the socio-economic environment and geographical location has long been used to examine inequalities in health. Studies in different populations have also observed a greater risk of dying from an alcohol-related cause in urban areas [13,14]. One study argued that the impact of a potential tax increase is expected to be modified by such factors as disposable income, drinking culture, and the demand elasticity for alcohol among various population groups [11]. There is insufficient evidence to elucidate fully the effects of alcohol prices or taxes on measures of alcohol-related morbidity or mortality linked to demography. A better understanding of the spatial factors that reduce alcohol-related risk may help health policy-makers to set priorities and to make appropriate measure available to vulnerable populations. Using a spatial approach, Ethan and colleagues [14] examined demographic differences in alcohol retail density and concluded that the density of alcohol retailers was associated with poverty and race. A Scottish study used a geographical analysis to examine gender differences in alcohol-related mortality [15]. However, there have been no studies comparing temporal changes in geographical disparities in alcohol-attributed disease mortality (ADM).

Due to its low cost and given certain cultural traditions, the domestic rice spirit produced by the Taiwan Tobacco and Wine Monopoly Bureau (TTWMB) has always been the most popular alcoholic beverage in Taiwan (approximately 200 million bottles sold per year). In the 1990s, US industries continued to request that Taiwan lower tariffs on imports of many goods, including alcohol. To comply with its World Trade Organization (WTO) commitments, in place of the previous tax on imports administered by the former monopoly authority, the TTWMB, Taiwan agreed to impose an excise tax and to eliminate tariffs on imports of most spirits. The TTWMB was then re-organized and became a state-owned corporation, i.e., the Taiwan Tobacco and Liquor Corporation (TTLC), in July 2002. As a condition of Taiwan’s WTO agreement, a new alcohol management and tax system went into effect on January 1, 2002. For example, rice alcohol tax rates increased from $0.7 per liter to $5.3 per liter at the time of the legislated change and then increased gradually to $6 per liter by 2003. Due to such taxes, the Rice Spirits retail prices were estimated to have increased sevenfold on average. Since 2002, the volume of rice spirits sold per year has decreased to 10 million bottles. Meanwhile, there has been no (or very little) change in tax rates for imported alcoholic beverages before or after 2002.

A recent study demonstrated that Taiwan’s tax policy may have a beneficial impact on alcohol-related chronic disease by changing drinking behaviors, but such influence differed according to sex and age [16]. In this study, we expected ADM rates to be generally lower after the change in tax policy due to decreased consumption. We also expected the reduction or increase of the mortality rate to vary with geographical area depending on specific life styles or social circumstances.

In this study, we used spatial analysis to explore whether spatial-temporal changes in the clustering patterns of ADM existed before and after the new tax policy was implemented. Our main focus was on geographical variations in alcohol-related mortality rates caused by the alcohol tax, not on the factors which might raise or lower the rate. We also did not attempt to identify the risk factors associated with higher alcohol-related mortality in some areas rather than others. Instead, we explored whether the effects of the tax on alcohol-related mortality rate were the same in different areas of Taiwan. Rather than the analysis of alcohol-related causes of death, the study emphasized ADM as the end-point of this study to reflect the direct influence of the alcohol tax. The mortality data covered two 5-year periods (1997 to 2001 and 2002-2006) for the determination of temporal changes.

## Methods

### Study area and spatial unit of analysis

In this study, we sought regions that were physically small enough to reflect spatial variations in mortality rates across Taiwan but were large enough when it comes to population to provide robust ADM estimates. Township level was used as the spatial unit of analysis for investigating geographical inequalities in mortality. As shown in Table 1, the township level is small enough for identifying areas across Taiwan with different urbanization levels and regions and determining differences in demographic and socioeconomic conditions. Therefore, geographical variations in population density and socioeconomic conditions across Taiwan can be captured at this spatial unit. In 1997–2001, Taiwan had 359 townships of different urbanization levels measuring 8–120 square kilometers and having an average population of 62,543 (0.1–20.7 thousand people per square kilometer). Each township had 3,700–60,000 households (Table 1(a)). Moreover, township is the basic unit of master plans in Taiwan, including healthcare resource plans, medical emergency zoning plans, and central governmental budget allocation projects. Most highly urbanized townships are concentrated in northern Taiwan and most of eastern townships are areas traditionally inhabited by aboriginal peoples with historical and cultural aboriginal characteristics, who are socioeconomically deprived compared to the general population (Table 1(b)).

**Table 1:** Township-level social-demographic variables summarized by (a) urbanization levels and (b) regions in Taiwan

	**Counts of townships (N)**	**Social-demographic variables**						
		**Average size**^**1**^	**Average population density**^**2**^	**Average number of households**^**3**^	**Sex ratio**^**4**^	**% of people with higher education**	**Personal income**^**5**^	**% of aboriginal peoples**
(a). Urbanization levels								
Highly-urbanized	27	8.2(13.76)	20.7(7.66)	59.1(4.71)	97.7(4.71)	33.3(9.09)	844(212)	0.4
Medium-urbanized	42	32.6(25.43)	4.6(3.19)	44.0(30.5)	100.8(3.12)	29.3(5.17)	749(117)	0.9
Boomtown	57	37.0(24.65)	1.6(1.67)	20.4(15.9)	106.0(3.18)	21.6(3.31)	634(64)	1.5
General towns	87	58.9(45.60)	0.6(0.61)	9.8(8.1)	109.0(4.81)	17.8(3.47)	593(61)	1.5
Ageing towns	35	72.9(43.39)	0.2(0.16)	3.7(2.9)	116.5(6.54)	11.5(2.35)	540(42)	3.9
Rural towns	61	119.8(267.00)	0.1(0.21)	5.0(2.9)	115.9(5.22)	12.3(2.85)	548(46)	21.8
Remote towns	40	75.8(332.86)	0.3(0.470	4.9(4.4)	111.3(6.32)	13.9(4.00)	555(59)	32.8
**Total**	**359**	**54.23(182.17)**	**0.67(0.58)**	**9.8(26.7)**	**108.9(7.89)**	**17.2(8.41)**	**589(133)**	**1.9**
(b). Regions								
North	89	82.62(122.14)	1.55(8.2)	21.44(39.1)	105.6(9.0)	23.2(10.0)	661(184)	0.8
Middle	106	99.12(209.32)	0.75(4.1)	9.65(14.7)	109.8(7.1)	17.0(6.5)	575(85)	0.3
South	135	74.64(98.04)	0.54(5.0)	8.86(20.9)	108.9(6.6)	16.4(7.3)	586(100)	0.2
East	29	280.82(358.29)	0.09(0.7)	3.39(9.6)	116.9(7.4)	10.5(5.1)	516(90)	38.0

(Source: Population and Housing Census in Taiwan-Fukien Area of the Republic of China, 2000)

Note: The numbers within the parentheses are standard deviation of social-demographic variables.

1. unit: Km^2^.

2. unit: thousand persons/km^2^.

3. unit: thousand.

4. Female = 100.

5. unit: thousands NT dollars. The data is acquired from the 2001 Income Tax Statistics released by the Ministry of Finance, Taiwan Central Government.

Mortality data from 1997 to 2006 were retrieved from Taiwan’s Death Registry (TDR). In Taiwan, causes of death are determined by the physicians who provided treatment or care to the decedent, and the death certificates are then confirmed by a coroner or pathologist. Deaths are classified according to the 9^th^ edition of the International Classification of Diseases (ICD-9). The accuracy and completeness of the TDR over the past decades in Taiwan have been considered adequate [17]. Information on sex, age at death, cause of death, place of death, and residential district (name and code of township) was obtained from death certificates. Alcohol-attributable diseases were defined based on comprehensive reviews of the literature [10,18]. Using ICD-9 codes, we counted individuals who died from ADM, including alcoholic liver disease (ICD code = 571.0-571.3), alcohol psychoses (291.0-291.9), alcohol abuse, alcohol dependence syndrome (303, 305.0), alcoholic polyneuropathy (357.5), alcoholic cardiomyopathy (425.5), alcoholic gastritis (535.3), and acute alcohol poisoning (980.0).

### Calculation of mortality rates

Individual-level mortality data were obtained, and the residence of each case was geocoded based on the name/code of township appearing on the TDR. Subsequently, data on the number of deaths in all townships were aggregated and further stratified by age group for the whole study area. We classified the study decade into two 5-years groups, 1997-2001 and 2002-2006, because alcoholic beverage taxation began in 2002. The annual mortality data were aggregated; the average number of deaths in the two 5-years periods was used as the numerators, and the household population in 2002 obtained from Taiwan’s household registrations as the denominator. The mortality data of the two periods were grouped to match the age structure of household data in 2002. Then, we calculated the raw mortality rate for each administration tract. We produced the direct, age-standardized mortality rate (DSR) for ADM based on the WHO’s world standard age-specific population [19]. The DSR of each township was mapped with ArcGIS 9.3 (ESRI Corp, Redlands, CA, USA) for the comparison of the spatial-temporal changes between 1997-2001 and 2002-2006. The rate ratio (RR) of ADMs was defined as the ratio of the DSRs in 1997-2001 to those in 2002-2006. If the RR value of a township is larger than 1, it indicates that there was a decline in DSR in that township, which suggests a beneficial effect of tax policy on ADM; a RR value less than 1 signifies the opposite. Moreover, the average population density of Taiwan in 1997–2002 and 2003–2006 is 611.8 (interquartile interval = 238.0-1748.8) and 628.1 (interquartile interval = 232.1-1818.9) people per square kilometer, respectively. The difference in population density between the two periods is not statistically significant (*p*=0.23), suggesting that there is no significant demographic transition between these two periods. RRs in all townships were measured to map geographical variations in temporal changes and thereby assess the effect of the new tax policy.

### Spatial analyses

We identified statistically significant spatial clusters of DSRs among townships by using G_i_*(d). Getis and Ord’s local spatial statistic G_i_*(d) was developed to determine local clustering [20]:

(1) $$G_{i}^{*}\left(d\right)=\frac{\sum_{j=1}^{n}w_{i,j}\left(d\right)x_{j}-\bar{X}\sum_{j=1}^{n}w_{i,j}\left(d\right)}{S\sqrt{\frac{n\sum_{j=1}^{n}w_{i,j}{\left(d\right)}^{2}-{\left(\sum_{j=1}^{n}w_{i,j}\left(d\right)\right)}^{2}}{n-1}}}$$

where G_i_*(d) is the spatial concentration statistic for township *i* and *d* is neighborhood threshold distance. The variable *x*_*j*_ is the DSR in a particular township *j*, and *w*_*ij*_ is a spatial weighting matrix, which measures the proximity of township *i* to township *j*. A value of 1 means that the distance between townships *i* and *j* is within the neighborhood threshold distance. Furthermore, $\bar{X}$ and S are the mean and standard deviation of *x*_*j*_, respectively. The mathematical formulas are as follows:

(2) $$\overset{―}{X}=\frac{\sum_{j=1}^{n}x_{j}}{n}$$

(3) $$S=\sqrt{\frac{\sum_{j=1}^{n}x_{j}^{2}}{n}-{\left(\overset{―}{X}\right)}^{2}}$$

The G_i_*(d) statistic is also a z-score, so its value is larger (smaller) if high (low) DSR values cluster together. The null hypothesis is that G_i_*(d) is equal to zero, suggesting that DSR values are distributed spatially without a defined pattern or are randomly distributed. In this paper, the level of significance for the G_i_*(d) value of DSR was set at 95%. Therefore, DSR hotspots can be identified when the *G*_i_*(d) value of township *i* is larger than 1.96.

## Results

### Spatial patterns of DSR

**Figure 1:**
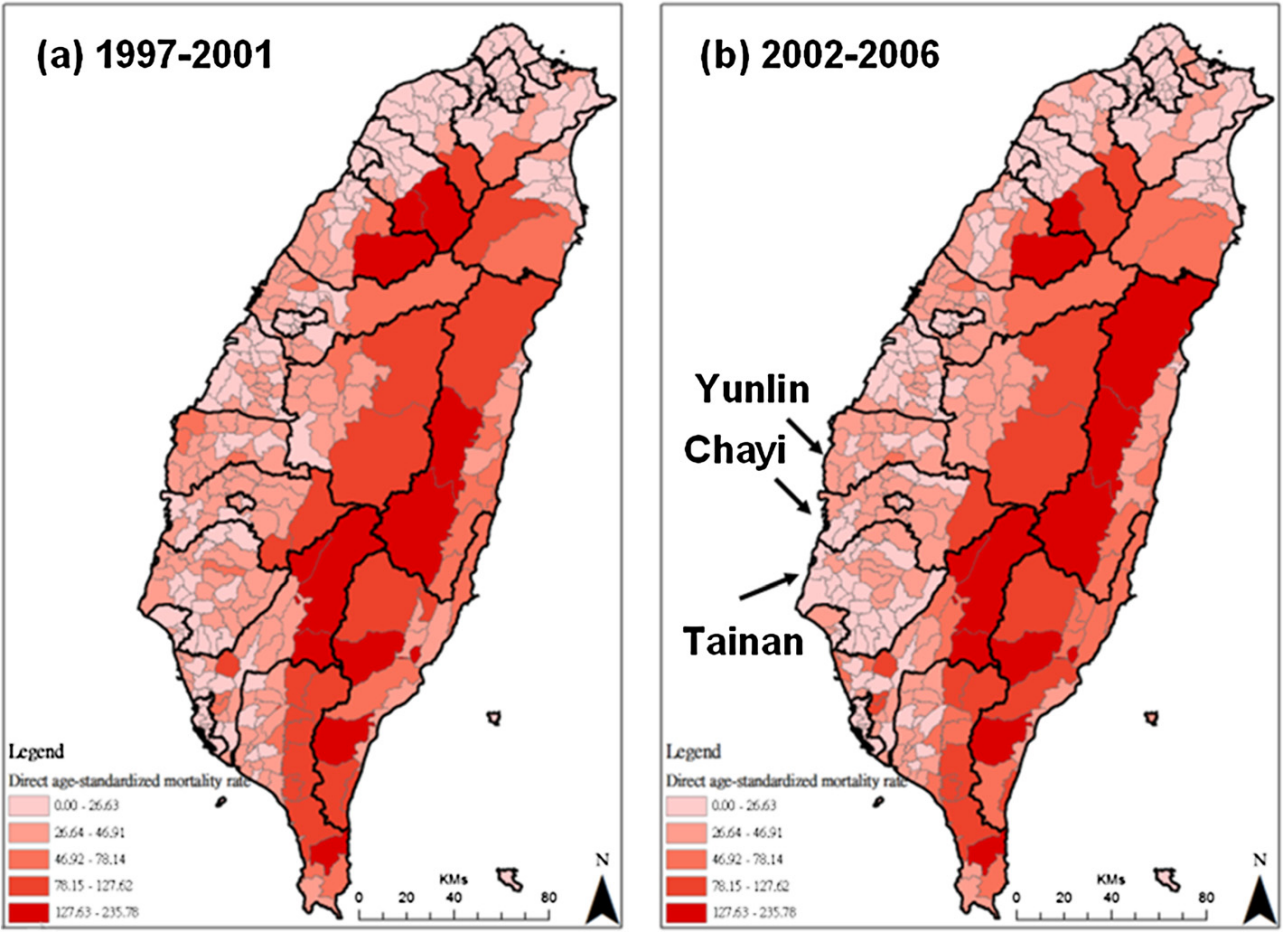
**Spatial distribution of direct age-standardized mortality rate (DSR) in different periods.** Panel (**a**) is the period: 1997-2001 and panel (**b**) is the period: 2002-2006.

**Figure 2:**
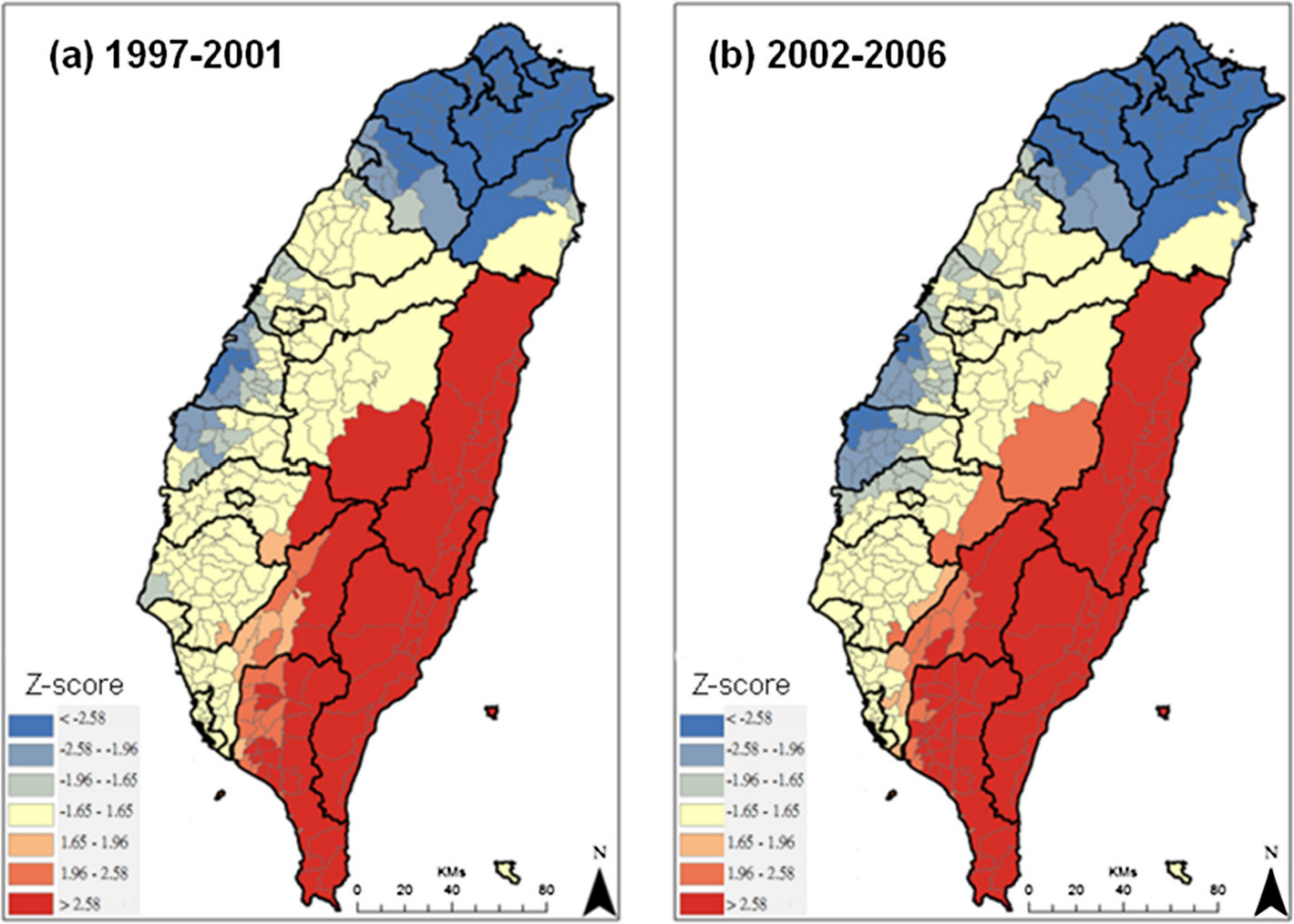
**Spatial concentration of direct age-standardized mortality rate (DSR) in different periods.** Panel (**a**) is the period: 1997-2001 and panel (**b**) is the period: 2002-2006.

The spatial distributions of DSR in the two periods, 1997-2001 and 2002-2006, are shown in Figure 1. The areas with higher alcohol disease mortality rates (127-235 deaths per 100,000 people) are located in eastern Taiwan, which consists mostly of mountainous areas. In northern Taiwan, which contains the most populated areas, the morality rates were lower (less than 26 deaths per 100,000 people) in both periods. The DSRs in the central and southwest areas, including Yunlin, Chayi and Tainan counties, became lower after 2002. Figure 2 shows spatial concentrations of DSR in both periods. Clusters of areas with higher DSRs and statistically significant clusters with high DSRs (z-score larger than 1.96) are shown in red. Meanwhile, clusters of areas with lower DSRs and statistically significant clusters with low DSRs (z-score less than -1.96) are shown in blue. The spatial patterns do not show significant temporal changes in the areas with the highest and lowest DSRs. The areas with lower DSRs in the central areas become larger and more concentrated.

### Spatial patterns of DSR ratios

**Figure 3:**
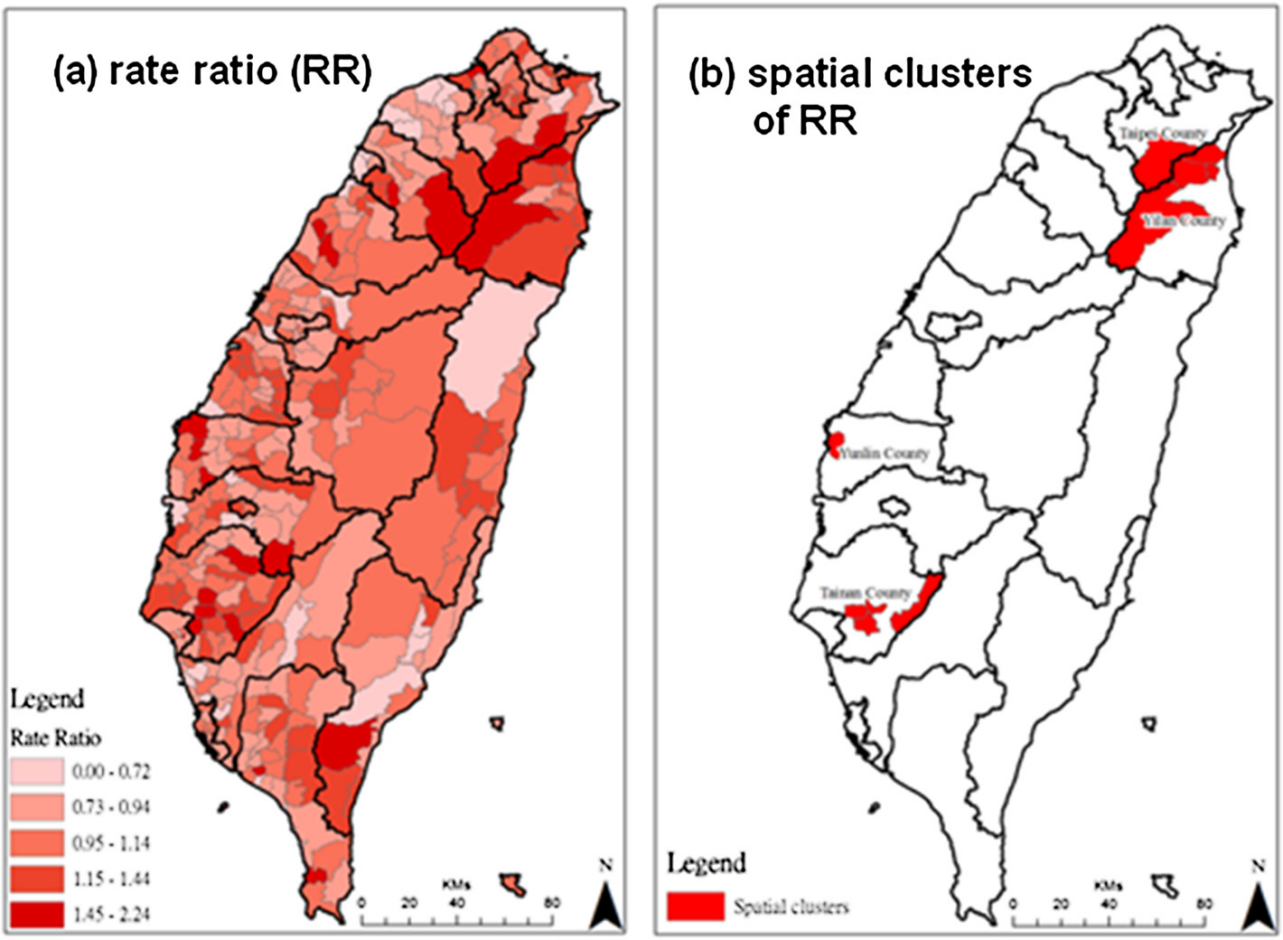
**Rate ratios (RR) of direct age-standardized mortality rate (DSR) between period 1997-2001 and 2002-2006.** Panel (**a**) shows spatial distribution of DSR rate ratio, and panel (**b**) shows spatial clusters of RR.

DSR ratios can indicate the trend of temporal changes between two periods. Spatial distributions and clustering of rate ratios are shown in Figures 3a and 3b. The areas with higher RRs are located in the southern, eastern and southwestern areas. These areas are sporadically distributed; therefore, these areas were not identified as having significant clusters of RRs. The areas where DSR significantly declined (RR > 1.5) are highlighted in red in Figure 3b, and these areas are clustered in the central, southwest and northeast areas, including parts of Yunlin, Chiayi and I-lan counties.

## Discussion

We found that townships with high ADM rates tend to cluster in remote areas. Similar to our findings, Blomgren et al. [21] indicated that there are lower levels of alcohol-related mortality in urban areas than in rural areas after adjustment for individual-level characteristics. In contrast, some studies in different populations have noted a greater risk of dying from acute or chronic alcohol-related causes in urban areas [13,14,22]. In addition to individual characteristics, the level of alcohol-related problems may vary with socio-economic status. For example, inequalities in access to healthcare are known to exist for many illnesses, with socio-economically disadvantaged individuals receiving different interventions [14]. Further investigation into such inequalities in relation to alcohol-related disorders is needed. From the results of the spatial analysis of distribution, reduction of ADM caused by alcohol taxation in response to international trade liberalization was not universal in Taiwan. Clusters of DSR reduction were observed in three independent pockets (as shown in Figure 3b). This study is an extension of recent work that found that implementation of the 2002 alcohol tax policy was followed by a reduction of ADM [16]. That the benefits of taxation varied with geographical area may reflect that they are related to specific life or social circumstances.

It is well known that chronic drinking may cause death from organ damage, such as liver cirrhosis [23,24]. Few (approximately 3%) of the specific causes of death included in our outcome measures were the result of the acute toxic effects of ethanol ingestion (e.g., poisoning), but most (over 85%) of the specific causes of death were chronic conditions that resulted from decades of high exposure to ethanol (e.g., alcoholic hepatitis or fatty liver). According to our results, the areas with high reductions in ADM coincided closely with the spatial pattern of high chronic hepatitis prevalence. Response to a change in drinking levels because of high price was more acute in the places where chronic hepatitis was prevalent. Rosenberg et al. suggested that people with severe mental illness, who exhibit elevated rates of both HBV and HCV and who also have a very high lifetime prevalence of alcohol use disorders, are at an unusually high risk for developing severe liver disease [25]. In addition, Erskine et al. [14] also implied that socio-economically deprived heavy drinkers are more likely to get serious liver disease. Mortality caused by long-term, chronic alcohol use responds immediately to a change in drinking levels because at any given time there is a reservoir of individuals in the population who are about to die from a chronic alcohol-related disease [23]. Even modest reductions in current drinking retards the progression of alcohol-related disease in this population, resulting in a reduction in the death rate, as was found in the present study.

The present study also identified clusters of ADM in central and eastern Taiwan (as shown in Figure 2), where the aboriginal population is dominant (Table 1); enhanced drinking prevention and control measures and efficient allocation of public health resources are required in these regions. 1.9 percent of the Taiwanese populations of 23 million people are of aboriginal ethnic groups, and they reside mainly in the central and eastern valleys (Table 1). One study in Taiwan demonstrated disparities in health between aboriginal and non-aboriginal individuals in a given population [26]. Another investigation showed a 60% prevalence of adult drinking among American Indians of the northern US states [27]. Drinking is also a traditional aboriginal habit during daily activities, festivals and ceremonial rituals in Taiwan. Previous studies have indicated that the prevalence of alcohol drinking increases with age, is higher among aborigines than among persons of Chinese origin, and is higher among those with lower levels of education than among those with higher levels [28,29]. As a specific cultural habit, the illegal distilling and consumption of rice spirits is popular among aboriginal tribes in Taiwan and that may increase the risk of impure alcohol consumption, resulting in higher rates of ADM. However, due to such illicit production, increasing alcohol prices may not affect consumption, which could diminish the benefit of alcohol taxation on ADM in this population.

The assumption that the price elasticity of alcoholic beverages entails that an increased tax on alcoholic beverages will raise the price paid by consumers, who respond by purchasing and drinking less alcohol [30,31]. One econometric analysis concluded that making alcohol more expensive and less available and banning alcohol advertising are highly cost-effective strategies to reduce alcohol-related harm [32]. Spatial analysis is obviously of value, and such methods will gradually become an integral component of epidemiological research and policy assessment. To our knowledge, there have been no national geographic studies assessing the effects of the tax policy imposed on alcohol-related diseases. Wagenaar and colleagues concluded that the size of the alcohol-tax effect is even more noteworthy given that state tax policy affects the entire population of a state, rather than the relatively small numbers of individuals [10]. Our results suggest that the impact of alcohol tax policy among different groups may vary, and this requires further analysis. Using the sensitivity map complemented with cluster detection, public health policymakers could better prioritize the specific areas where comprehensive investigations should be undertaken.

Although complete and accurate measures of the intervening factor (i.e., drinking behavior) were not available in our study, evidence concerning the impact of alcohol taxes on the rate of ADM presented in our study is so well-established that a lack of measures for intervening factors does not affect the plausibility of the findings. Moreover, alcohol taxes may decrease the intention to purchase alcohol in legal markets, providing an incentive to pursue illicit alcohol consumption and increasing the possibility of alcohol-related disease and death. Unfortunately, we were unable to take into account the influence of illicit alcohol consumption in this study because the data were almost impossible to obtain. However, such methodological imperfection should not be considered a valid argument against our findings because our results showed significant declines rather than increases in rate of ADM after the implementation of the alcohol tax policy. Although it could be argued that other underlying factors, such as obesity or other chronic diseases may influence the relationship between drinking and alcohol-related mortality [33,34], those factors may be not associated with alcohol taxation. These factors would not confound the temporal changes between the two periods in this study.

Our study has some limitations. First, following most researchers dealing with geographical units, we used an administrative unit (township) as spatial unit of analysis and geometric relationship (distance-based contiguity) as the definition of proximity. However, township-level areas have little substantive meaning when it comes to delineating neighborhoods, communities, or cultural boundaries, leading to difficulties in precisely describing geographical proximity and the extent to which appropriate boundaries may be delineated. Second, our results showed township-level geographical disparities across the whole of Taiwan, but we note that the township level is also a relatively large administrative unit for local public health practices. Each township in Taiwan is composed of 25–50 villages, so the township level may mask important variations at the village level or even at the community/neighborhood level [35]. Lastly, taxes for other alcoholic beverages became less (or not adjusted) than that for rice spirits after 2002. Thus, the new tax policy may affect only those consuming rice spirits, not the whole population. Despite the above limitations, this study still provides significant evidence that a township-level relationship between the reduction of ADM and alcohol taxation exists in Taiwan. In those areas that have a high prevalence of chronic liver disorders, the alcohol tax policy may also have a more beneficial impact on alcohol-related disease by changing drinking behaviors.

## Conclusions

This study provides the evidence of a township-level association between the reduction of ADM and alcohol taxation in Taiwan. The reduction of the mortality rate varies with geographical area. . The significant association pockets of ADM tend to cluster in remote townships, where the aboriginal population is dominant. It may be associated with changes of specific life styles.

## Competing interests

The authors have declared that no competing interests exist.

## Authors' contributions

CML and THW designed the research and wrote the paper. THW generated the figures and the table. Both authors analyzed the data, interpreted the results, and contributed to the discussion. All authors read and approved the final manuscript.

## Pre-publication history

The pre-publication history for this paper can be accessed here:

http://www.biomedcentral.com/1471-2458/12/889/prepub
